# Self-supervised learning and transformer-based technologies in breast cancer imaging

**DOI:** 10.3389/fradi.2025.1684436

**Published:** 2025-11-07

**Authors:** Lulu Wang

**Affiliations:** 1 Department of Engineering, Reykjavik University, Reykjavik, Iceland; 2 College of Science, Engineering and Technology, University of South Africa, Midrand, South Africa

**Keywords:** breast cancer, self-supervised learning, transformers, medical imaging, artificial intelligence

## Abstract

Breast cancer is the most common malignancy among women worldwide, and imaging remains critical for early detection, diagnosis, and treatment planning. Recent advances in artificial intelligence (AI), particularly self-supervised learning (SSL) and transformer-based architectures, have opened new opportunities for breast image analysis. SSL offers a label-efficient strategy that reduces reliance on large annotated datasets, with evidence suggesting that it can achieve strong performance. Transformer-based architectures, such as Vision Transformers, capture long-range dependencies and global contextual information, complementing the local feature sensitivity of convolutional neural networks. This study provides a comprehensive overview of recent developments in SSL and transformer models for breast lesion segmentation, detection, and classification, highlighting representative studies in each domain. It also discusses the advantages and current limitations of these approaches and outlines future research priorities, emphasizing that successful clinical translation depends on access to multi-institutional datasets to ensure generalizability, rigorous external validation to confirm real-world performance, and interpretable model designs to foster clinician trust and enable safe, effective deployment in clinical practice.

## 1 Introduction

Breast cancer is the most common malignancy among women worldwide and remains a leading cause of cancer-related mortality (1). Early and accurate detection is essential for guiding treatment decisions, improving clinical outcomes, and increasing patient survival (2). Medical imaging modalities, including mammography, ultrasonography, and magnetic resonance imaging (MRI), play critical roles in screening, diagnosis, and monitoring of breast lesions.

Mammography is the established gold standard for population-based breast cancer screening due to its cost-effectiveness and high spatial resolution, which enables early detection of malignancy indicators such as microcalcifications and architectural distortions (3). Its effectiveness is bolstered by standardized protocols and widespread availability, supporting its central role in early detection programs globally. However, mammography relies on low dose ionizing radiation, raising concerns about cumulative exposure, and requires breast compression, which can cause patient discomfort. More importantly, its sensitivity decreases significantly in women with dense breast tissue, where overlapping fibroglandular tissue can obscure lesions and increase false-negative rates.

To address these limitations, breast ultrasonography is often used as a complementary modality, especially in women with dense breasts (4). Ultrasound offers real-time imaging without ionizing radiation and effectively differentiates cystic from solid lesions. However, its diagnostic accuracy is highly operator-dependent, and variability in image quality can lead to inconsistent interpretations.

MRI serves as an advanced adjunctive tool, particularly valuable for high-risk populations and preoperative staging (5). It offers superior soft-tissue contrast and enhanced sensitivity for detecting invasive cancers, especially in dense breast tissue. Contrast-enhanced MRI can also visualize angiogenic activity associated with malignancy. Despite these advantages, broader clinical adoption remains limited by high cost, limited accessibility, lengthy examination times, and concerns regarding the safety of gadolinium-based contrast agents.

Artificial intelligence (AI), particularly convolutional neural networks (CNNs), has recently attracted significant attention in breast imaging, with applications in lesion segmentation, classification, detection, and risk stratification (6–8). Encoder–decoder architectures such as U-Net preserve fine-grained spatial information through skip connections (9), while deep residual networks (10) and densely connected networks (11) address vanishing gradient issues, enabling more effective hierarchical feature extraction. A key limitation of conventional supervised CNNs, however, is their reliance on large annotated datasets, which are costly to produce and often restricted by privacy regulations. To address this challenge, self-supervised learning (SSL) has emerged as a label-efficient strategy that can be applied across CNNs, transformers, and hybrid architectures (12). SSL uses surrogate tasks such as inpainting, context prediction, rotation, or contrastive learning on unlabeled data to pretrain models on unlabeled data. These pretrained models can then be fine-tuned on smaller labeled datasets, achieving competitive downstream performance while reducing annotation requirements.

Recently, Transformer-based models, particularly Vision Transformers (ViTs), have been adapted for breast imaging to overcome the limited receptive fields of CNNs (13). By employing multi-head self-attention mechanisms, ViTs capture long-range dependencies and global contextual information, which are especially valuable for detecting diffuse lesions and subtle architectural distortions. Although attention maps from ViTs can highlight image regions that influence predictions, similar interpretability can also be achieved in CNNs through saliency-based methods such as Grad-CAM and SHAP. Hybrid architectures that integrate CNN backbones with transformer modules have demonstrated synergistic performance, combining the local feature sensitivity of convolutional layers with the global contextual reasoning enabled by self-attention (14). This fusion represents a promising direction for advancing AI-driven breast cancer imaging.

This study reviews recent advances in SSL and Transformer-based approaches in breast imaging, addressing key tasks including segmentation, detection, and classification across mammography, ultrasound, and MRI. The study also addresses current challenges and limitations, including annotation scarcity, image heterogeneity, and barriers to clinical implementation. The following research questions are addressed:

1. How can SSL pretraining strategies be optimized to learn robust and transferable feature representations from unlabeled breast imaging datasets, thereby reducing reliance on large-scale labeled data?
2. Which self-supervised pretext tasks most effectively enhance downstream performance in breast imaging applications—such as lesion detection, segmentation, and classification—across diverse imaging modalities?
3. To what extent do ViTs and hybrid CNN–Transformer architectures capture global contextual information in multi-modal breast images, and how does this impact diagnostic accuracy compared to purely convolutional models?
4. Can integrating SSL with Transformer-based architectures mitigate modality-specific artifacts and improve cross-modal data harmonization, thereby enabling more consistent and reproducible AI-driven interpretations?
5. What practical and regulatory challenges must be addressed to implement SSL–Transformer frameworks in routine clinical workflows, particularly regarding model interpretability, computational efficiency, and clinician adoption?

The remainder of this paper is organized as follows. Section 2 presents the materials and methods applied in this study. Section 3 provides an overview of foundational SSL, Transformer, and hybrid architectures. Sections 4 through 6 present application-specific findings in segmentation, detection, and classification, respectively. Section 7 discusses current limitations, clinical implications, and future research directions. Finally, Section 8 concludes the review.

## 2 Materials and methods

This study offers a summary of key research that uses SSL and transformer-based models in breast cancer imaging. Its goal is to provide an overview of current methods, datasets, types of supervision, reported results, as well as the main challenges and limitations.

### 2.1 Eligibility criteria

Peer-reviewed studies were considered eligible if they applied SSL or transformer-based architectures to breast cancer imaging tasks, including classification, detection, or segmentation.

- Definition of SSL models: In this review, SSL refers to approaches in which model representations are learned from unlabeled data through pretext tasks (e.g., contrastive learning, masked image modeling, clustering-based objectives, rotation prediction, or inpainting) prior to fine-tuning on downstream diagnostic tasks.
- Supervised transformers: Supervised transformer models were included only when directly relevant to breast imaging tasks, to contextualize the broader adoption of transformer architectures.
- Exclusion criteria: Studies were excluded if they (i) employed only conventional supervised convolutional neural networks (CNNs) without SSL or transformer components, (ii) did not report empirical results on breast imaging datasets, or (iii) were reviews, editorials, or conference abstracts lacking full data.

### 2.2 Information sources and search strategy

A literature search was conducted in PubMed, Scopus, IEEE Xplore, and Web of Science for English-language publications published between January 2015 and June 2025. Boolean logic was used to combine three conceptual domains: breast cancer pathology, imaging modalities, and machine learning approaches. The search strategy included terms such as (“breast cancer” OR “breast tumor” OR “breast lesion”) AND (“mammography” OR “ultrasound” OR “MRI”) AND (“self-supervised learning” OR “SSL” OR “representation learning”) AND (“transformer” OR “vision transformer” OR “ViT” OR “attention mechanism”).

### 2.3 Study selection

All retrieved records were imported into EndNote, and duplicates were removed. Titles and abstracts were then screened for relevance, followed by full-text review. As this is a narrative review, the screening process was conducted by the author. In total, 761 records were identified: 155 from PubMed, 249 from Scopus, 249 from IEEE Xplore, and 108 from Web of Science. After duplicate removal and relevance screening, 137 full-text articles were assessed, of which 85 met the inclusion criteria.

### 2.4 Data extraction and synthesis

Key study characteristics were extracted, including the authors, year of publication, imaging modality, dataset size and source, supervision regime, external validation status, model type, downstream task (classification, detection, or segmentation), and reported performance metrics (e.g., AUC, Dice, sensitivity, specificity). Where available, comparisons with baseline methods were also recorded. The data were synthesized narratively and summarized in structured tables (Tables 1–3). Missing information was indicated as “NR” (Not Reported).

**Table 1:** SSL-based breast image segmentation.

Refs	Year	Model/Framework	Imaging modality	Task	Dataset(s) & sample size	Supervision regime	External validation	Performance results	Relative improvement vs. baseline
(15)	2021	BYOL-based Self-Supervised Visual Transformers	Mammography	Lesion segmentation & diagnosis	1,227 mammograms (UMC Hospital, Kazakhstan)	SSL vs. supervised baseline	NR	IoU ↑ ∼4%, Val. Acc ↑16.7%, Loss ↓12.5%	Moderate gain
(16)	2021	CR-SSL (Closely Related Self-Supervised Learning)	Ultrasound	Tumor segmentation	BUS dataset (780 images)	SSL vs. supervised CNN	NR	Dice 73.0–82.1%, Jaccard 70.8–80.1%	+10%–20% Dice/Jaccard
(17)	2025	DIGN + APMN + STA-UNet	Ultrasound	Tumor segmentation	Mendeley, SIIT (sample sizes NR)	SSL-enhanced Transformer	NR	Dice ↑2%–4%, HD ↓30%–38%	Small–moderate gain
(18)	2025	CT-Match (semi-supervised CNN + Transformer)	Ultrasound	Semi-supervised tumor segmentation	Dataset A: 780 images (600 pts, Baheya Hospital); Dataset B: 163 BUS images	Semi-supervised (SSL + supervised)	NR	Dice 78.9% (A), 84.9% (B)	Outperformed supervised baselines
(19)	2025	Global Content Perception + Peritumoral Context Restoration	DCE-MRI	Tumor segmentation	229 MRI cases	SSL vs. semi-supervised baselines	NR	Dice 87.8%, HD95 7.47 mm	Outperformed 9 SSL/semi-sup baselines
(20)	2025	QMaxViT-Unet + (Query-based MaxViT-Unet with Edge Enhancement)	Multi-modal (incl. BUSI)	Scribble-supervised segmentation	ACDC, MS-CMRSeg, SUN-SEG, BUSI	Weakly/scribble-supervised	NR	Dice 69.4% (BUSI), others 71%–89%	Competitive with dense labels

**Table 2:** SSL-based breast image classification.

Refs	Year	Model	Imaging modality	Task	Dataset(s) & sample size	Supervision regime	External validation	Performance results	Relative improvement vs. baseline
(21)	2024	DSMT-Net (Dual Self-Supervised Multi-Operator Transformation)	Endoscopic Ultrasound (EUS) and Breast Ultrasound	Pancreatic vs. non-pancreatic; Benign vs. malignant breast lesions	LEPset: 3,500 labeled + 8,000 unlabeled EUS; BUSI: 780 BUS images	SSL pretraining + supervised fine-tuning	Yes (BUSI)	LEPset: Acc 87.7%, F1 82.2%; BUSI: Acc 89.2%, F1 88.1%	+5%–7% Acc vs. supervised CNN
(22)	2025	BCT-Net (CNN + Transformer with SCCA)	BUS	Hybrid: segmentation + classification	BUSI: 780 images (3 classes)	SSL contrastive alignment	NR	Precision 86.1%, Dice 88.7%	Outperformed CNN baseline
(23)	2025	Flip Learning (Multi-Agent RL + Curriculum)	2D BUS, 3D ABUS	Hybrid: nodule segmentation + classification	BUS: 2,401 train, 338 val, 680 test-A, 1,230 test-B; ABUS: 1,124 train, 125 val, 486 test	Weakly supervised SSL	NR	BUS Dice 92.4%; ABUS Dice 75.5%	Comparable to fully supervised with fewer labels
(24)	2021	Self-/Weakly Supervised Autoencoder	Mammography	Abnormality detection & classification	INBreast; private multi-vendor	SSL + weak supervision	Yes (multi-vendor)	AUC 86%; TPR 93%; F1 64%	Improved vs. supervised autoencoder
(25)	2025	AGE (Attention-Guided Erasing, DINO-based SSL)	DM, CEM, SFM	Density, malignancy, calcification classification	Multiple mammography datasets	SSL pretraining	NR	F1 ↑2% (DM), ↑1.5% (CEM), ↑0.4% (SFM)	Marginal gains
(26)	2025	PatchCascade-ViT	Mammography	BI-RADS classification	4,368 mammograms	SSL ViT	NR	Sensitivity 85.0%; F1 84.9%	Outperformed CNN baselines
(27)	2025	CascadePLS-ViT	Mammography	BI-RADS classification (3 classes)	4,368 mammograms	SSL ViT	NR	Recall 85.0%; F1 84.9%	Similar to (30)
(28)	2022	Semi-supervised ViT (ATS + CT)	BUS + Histopathology	Breast cancer classification (3-class BUS; 2-class BreakHis)	BUSI; BreakHis: 7,909 images (2,480 benign, 5,429 malignant)	Semi-supervised ViT	Yes (BreakHis)	BUSI: Acc 95.3%, F1 96.2%; BreakHis: Acc 98.1%, F1 98.4%	+3%–5% Acc vs. supervised

**Table 3:** SSL-based breast image detection.

Refs	Year	Model	Imaging modality	Task	Dataset(s) & sample size	Supervision regime	External validation	Performance results	Relative improvement vs. baseline
(29)	2025	DATTR2U-Net (Double Attention Recurrent Residual U-Net with Multi-Task SSL)	ABUS	Mass detection	TDSCABUS public dataset (size NR)	SSL proxy tasks (rotation + reconstruction)	NR	Recall 79.6%; FP 5.67/volume	∼+6% recall vs. supervised baseline (reported)
(24)	2021	Two-Channel Autoencoder (Self- & Weakly Supervised)	Mammography	Abnormality detection (masses, calcifications, distortions)	INBreast; private multi-vendor dataset (size NR)	SSL + weak supervision	Yes (multi-vendor)	AUC 0.86; TPR 93%; F1 64% (malignant masses)	Improved vs. supervised autoencoder
(30)	2024	CNN with Weakly Supervised Saliency Mapping	Mammography	Dense tissue localization	RSNA Breast Cancer Detection (4,387 curated images)	Weak supervision (saliency maps)	NR	Accuracy 75.3%; Dice 75.4%	Comparable to supervised U-Net with ∼15% labels
(31)	2025	Modified YOLOv8 + BRA + BiFPN	Mammography	Calcification detection	Contrast-enhanced & standardized mammograms (size NR)	SSL pretraining + supervised detection	NR	Precision 99.3%; Recall 85.0%; F1 91.6% (IoU 0.6)	Higher precision vs. baseline YOLO
(32)	2023	SSRL (Self-Supervised Rotation Learning, ResNet50)	MRI	Binary classification (cancer vs. healthy)	Kaggle Breast MRI (1,480 images: 740 cancer, 740 healthy)	SSL proxy task (rotation)	NR	AUC 95.8%; Acc 92.5%; Sens 95%; Spec 90%	Outperformed ImageNet pretraining

## 3 Related work

### 3.1 Self-supervised learning frameworks

SSL has emerged as a transformative paradigm in machine learning, enabling models to learn meaningful feature representations from unlabeled data by exploiting intrinsic image patterns and structures (33). Unlike traditional supervised approaches that rely heavily on manual annotation, SSL employs surrogate or “pretext” tasks—such as predicting missing image regions, reconstructing corrupted inputs, or aligning multiple views of the same sample—to guide models toward learning semantically rich embeddings. By solving these tasks, SSL frameworks capture hierarchical features that transfer effectively to diagnostic applications, including classification, detection, and segmentation.

In medical imaging, SSL methodologies are commonly grouped into three categories: contrastive, non-contrastive, and generative. Contrastive methods (e.g., SimCLR, MoCo) encourage representation learning by maximizing agreement between differently augmented views of the same image while minimizing similarity to unrelated samples, typically using an InfoNCE loss. Non-contrastive approaches (e.g., BYOL, SimSiam) eliminate the need for negative pairs by employing dual-network architectures that align representations without momentum encoders. Generative strategies, such as denoising autoencoders and generative adversarial networks, aim to model the underlying data distribution by reconstructing or synthesizing data. These approaches often improve robustness to noise and help handle class imbalance, particularly for underrepresented lesion types.

In breast imaging, SSL-based models have been increasingly adopted to address challenges such as limited data availability, high annotation costs, and inter-observer variability. Contrastive learning has demonstrated improvements in lesion classification for mammography and ultrasound, enhancing generalization from relatively small datasets. Non-contrastive pretraining on unlabeled ultrasound data has produced robust feature encoders that require minimal fine-tuning for downstream tasks. Generative SSL techniques have been applied to tasks such as noise reduction and synthetic oversampling, improving classifier performance for rare lesion categories. Building on this foundation, Section 4 explores how SSL has been applied in breast imaging across classification, detection, and segmentation tasks.

### 3.2 Transformers

Transformers have revolutionized computer vision by modeling long-range dependencies through self-attention mechanisms (34). Unlike CNNs, which rely on local receptive fields and translation equivariance, transformers compute global relationships among all input elements, enabling richer contextual representation. In breast imaging, this capability is particularly advantageous for identifying lesions, subtle architectural distortions, and bilateral asymmetries.

Vision Transformers (ViTs) adapt the original Transformer architecture for image analysis by partitioning images into fixed-size patches, linearly embedding them, and processing the resulting sequence through stacked encoder layers (35). This design facilitates global context modeling from early stages but lacks the inductive biases of CNNs, such as locality and hierarchical feature extraction. Consequently, ViTs typically require large annotated datasets and significant computational resources—constraints that are especially pronounced in medical imaging.

To overcome these limitations, hierarchical variants such as the Swin Transformer have been developed (36). Swin Transformers divide images into non-overlapping windows and apply local self-attention, using a shifted-window mechanism to enable cross-window information flow. This approach reduces computational cost and supports multi-scale feature learning, making it suitable for high-resolution breast imaging tasks such as mass segmentation and tissue classification. Not all Transformer models are self-supervised. This review includes only architectures incorporating SSL pretraining—such as masked autoencoders, contrastive learning, or generative modeling—while excluding purely supervised variants to maintain scope consistency and avoid conflating learning paradigms.

Transformer-based frameworks have shown strong performance across breast imaging modalities. For instance, UNETR (37) and TransUNet (38) integrate Transformer encoders into U-Net–style segmentation frameworks, enabling precise lesion boundary delineation in MRI and ultrasound. T-SVDNet (39) exploits higher-order prototypical correlations for domain adaptation, improving generalization across heterogeneous datasets. Although attention maps can enhance model interpretability, comparable visualization techniques such as SHAP and Grad-CAM remain applicable to CNNs.

In summary, Transformer architectures offer powerful tools for breast imaging analysis, particularly when combined with SSL pretraining. Their capacity for global context modeling complements the local feature sensitivity of CNNs, and hybrid designs consistently outperform traditional architectures in segmentation, classification, and detection tasks. Nevertheless, challenges persist regarding data requirements, computational efficiency, and clinical integration—issues addressed in subsequent sections.

### 3.3 Hybrid models

Hybrid CNN–Transformer models are designed to leverage the complementary strengths of CNNs and Transformer-based architectures within a unified learning framework (36). They exploit the inherent inductive biases of CNNs—like translation equivariance and localized receptive fields—to efficiently extract low-level, hierarchical features while keeping computational costs low. Simultaneously, they incorporate the global context modeling strengths of Transformers, which are critical for capturing long-range dependencies and complex spatial relationships. This two-stage approach creates a representational synergy: CNNs encode fine-grained textures and structural patterns, while Transformers integrate information across the entire image.

In typical hybrid architecture, the initial stage comprises a deep CNN backbone that processes raw input images into multi-scale feature maps (37). The early convolutional layers capture local contrasts and edge features, which are particularly important for detecting small or subtle lesions. This early processing also reduces spatial resolution, helping to limit the computational cost of applying self-attention to high-resolution medical images. Once compressed, spatial regions are projected into fixed-dimensional token vectors, and positional embeddings are added to preserve spatial relationships.

The second stage involves one or more Transformer encoder blocks that process these tokenized representations. In each block, multi-head self-attention captures pairwise dependencies between tokens, enabling the model to prioritize features based on broader contextual relevance. This is particularly advantageous in breast imaging, where assessing bilateral symmetry and connecting distant yet clinically related regions is often essential. Layer normalization and feed-forward networks further refine these globally informed features, which can then be reconstructed into spatial maps to support downstream tasks such as segmentation, detection, and classification.

The integration of CNN and Transformer components varies across hybrid architectures. In sequential designs, CNNs first extract features, which are then processed by Transformers for global reasoning. This simplifies information flow but adds overhead during tokenization of CNN outputs. In contrast, dual-stream architectures process inputs simultaneously through separate CNN and Transformer branches, merging outputs via learned fusion mechanisms to reconcile local and global representations. While this enhances representational diversity, it also increases model complexity and training demands. A third approach embeds lightweight attention modules within CNN layers, expanding the receptive field without incurring the full computational cost of dedicated Transformer modules. This strikes a balance between efficiency and expressive power.

To further enhance performance in high-resolution breast imaging, recent developments have introduced localized and hierarchical attention mechanisms (38, 39). For instance, the Swin Transformer uses a shifted window mechanism, restricting self-attention to overlapping local regions (38).

This reduces attention complexity from quadratic to linear in relation to token count, making it feasible for megapixel-scale scans, such as digital mammography and MRI. Hierarchical attention frameworks further align with radiologists' workflows, aggregating information from small regions of interest to form a holistic view, mimicking a zoom-in/zoom-out interpretive process (39). Moreover, spatially adaptive tokenization allocates more tokens to diagnostically significant regions, directing computational resources where they matter most, enhancing both accuracy and efficiency.

## 4 Self-Supervised learning in breast imaging

**Figure 1:**
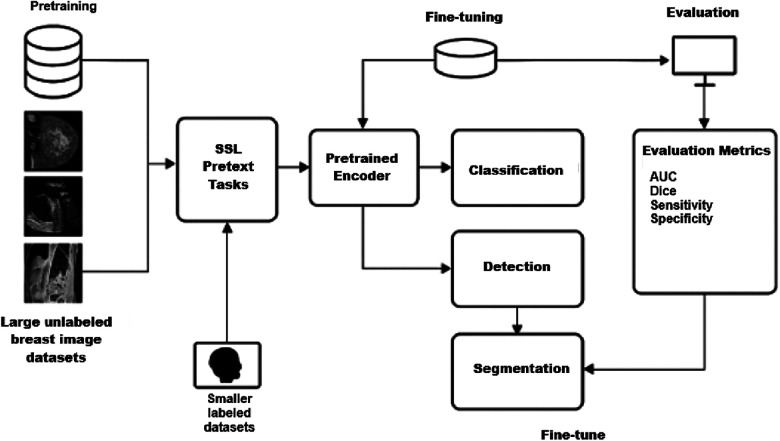
Representative SSL pipeline for breast imaging.

This section reviews the application of SSL frameworks to breast imaging tasks, including segmentation, classification, and detection. Figure 1 illustrates a representative SSL pipeline, showing how unlabeled data are exploited during pretraining and subsequently fine-tuned for specific diagnostic tasks. The following subsections provide a summary of the included studies, highlighting the employed model strategies, imaging modalities, specific tasks, datasets, and performance results.

### 4.1 SSL-based breast image segmentation

SSL has been increasingly applied to breast lesion segmentation across mammography, ultrasound, and MRI, aiming to reduce annotation burden while improving model robustness. Table 1 summarizes representative studies, including dataset characteristics, supervision regimes, and reported performance.

Early work by Saidnassim et al. (15) introduced a BYOL-based transformer for mammographic lesion segmentation. Using 1,227 mammograms from UMC Hospital (Kazakhstan), their SSL pretraining improved validation accuracy by 16.7% and reduced loss by 12.5% compared with supervised baselines, highlighting the potential of contrastive SSL in dense breast tissue.

In ultrasound imaging, Mishra et al. (16) developed Closely Related SSL (CR SSL), tailored to the low resolution and noise inherent in breast ultrasound. On the BUS dataset, CR SSL achieved Dice scores up to 82.1% and Jaccard indices above 80%, representing 10%–20% improvements over supervised CNNs. Similarly, Zhang et al. (17) combined SSL with transformer-based architectures (DIGN + APMN + STA-UNet), reporting Dice improvements of 2%–4% and Hausdorff distance reductions of 30%–38% on the Mendeley and SIIT ultrasound datasets.

Semi-supervised approaches have also been explored. El Sayed et al. (18) proposed CT Match, a dual-branch CNN–Transformer framework enforcing cross-architectural consistency. On 780 in-house ultrasound images and 163 BUS images, CT Match achieved Dice scores of 78.9% and 84.9%, respectively, outperforming supervised baselines despite limited labeled data.

In MRI, Meng et al. (19) introduced a dual-module SSL paradigm combining global content perception with peritumoral context restoration. Evaluated on 229 dynamic contrast-enhanced MRI cases, the model achieved a Dice score of 87.8% and HD95 of 7.47 mm, surpassing multiple SSL and semi-supervised baselines.

Weakly supervised SSL has also demonstrated promise. NguyenTat et al. (20) proposed QMaxViT-UNet+, a query-guided transformer trained with scribble annotations. Despite the absence of dense pixel-level labels, the model achieved a Dice score of 69.4% on the BUSI dataset and 71%–89% on other modalities, demonstrating strong label efficiency.

Collectively, these studies suggest that SSL can enhance segmentation accuracy and label efficiency across imaging modalities. However, the magnitude of benefit varies depending on dataset, supervision regime, and evaluation protocol. Many studies lacked external validation or standardized baselines, limiting direct comparison. Future work should prioritize multi-institutional datasets, consistent reporting of supervision regimes, and external validation to establish the generalizability of SSL-based segmentation methods.

### 4.2 SSL-based breast image classification

SSL has been increasingly applied to breast image classification tasks, encompassing mammography, ultrasound, and multimodal pipelines. By leveraging unlabeled or weakly labeled data, SSL frameworks aim to reduce annotation costs while improving generalization across heterogeneous imaging cohorts. Table 2 provides an overview of representative studies, highlighting the employed model strategies, imaging modalities, specific tasks, datasets, and performance results.

Li et al. (21) introduced DSMT-Net, a dual self-supervised multi-operator transformation framework that jointly leverages endoscopic ultrasound (EUS) and breast ultrasound (BUS) for lesion classification. Using 3,500 labeled and 8,000 unlabeled EUS images (LEPset) alongside 780 BUSI images, DSMT-Net achieved accuracies of 87.7% (EUS) and 89.2% (BUSI), with balanced precision and recall. Compared with supervised CNN baselines, SSL pretraining provided 5%–7% accuracy gains, demonstrating robust cross-domain performance.

Hybrid approaches combining segmentation and classification have also been explored. Xin et al. (22) proposed BCT-Net, integrating CNN and transformer modules with semantic contrastive alignment. On the BUSI dataset (780 images across three classes), the model achieved 86.1% precision and a Dice score of 88.7%, outperforming CNN baselines. Similarly, Huang et al. (23) developed Flip Learning, a weakly supervised SSL framework combining multi-agent reinforcement learning with curriculum learning. On 2D BUS and 3D ABUS datasets, Flip Learning achieved Dice scores of 92.4% (BUS) and 75.5% (ABUS), comparable to fully supervised methods despite using weaker labels, highlighting SSL's label efficiency in volumetric imaging.

In mammography, Tardy et al. (24) demonstrated that self- and weakly supervised autoencoders can achieve clinically relevant performance for abnormality detection. On INBreast and a private multi-vendor dataset, their model reached an AUC of 86%, a region-level true positive rate of 93%, and a pixel-wise F1 score of 64% for malignant masses, underscoring SSL's potential in multi-vendor settings. Panambur et al. (25) extended this approach with AGE, a DINO-based SSL framework with attention-guided erasing. Across digital, contrast-enhanced, and scanned-film mammography, AGE improved F1 scores by 0.4%–2% depending on the task, representing incremental but consistent gains.

Transformer-based SSL has also been applied to BI-RADS classification. Abdallah et al. (26, 27) introduced PatchCascade ViT and CascadePLS ViT, achieving sensitivity and F1 scores of approximately 85% on 4,368 mammograms, outperforming CNN baselines in both breast density and cancer risk categorization. These findings suggest that SSL-based ViTs can capture global context in high-resolution mammograms more effectively than conventional architectures.

Finally, multimodal SSL has been extended to cross-domain classification. Zhang et al. (28) proposed a semi-supervised ViT with adaptive token sampling and consistency training, evaluated on BUSI ultrasound and BreakHis histopathology datasets. The model achieved 95.3% accuracy on BUSI and 98.1% on BreakHis, representing 3%–5% gains over supervised baselines and demonstrating the transferability of SSL features across imaging domains.

Collectively, these studies indicate that SSL can enhance classification accuracy and label efficiency across modalities. However, the magnitude of improvement varies, with some methods yielding only marginal gains. Reporting gaps remain: external validation was inconsistently performed, and supervision regimes were not always clearly defined. Future work should prioritize multi-institutional datasets, standardized reporting of baselines, and rigorous external validation to establish the clinical utility of SSL-based classification methods.

### 4.3 SSL-based breast image detection

The scarcity of expertly annotated breast imaging datasets has accelerated the adoption of SSL and weakly supervised learning (WSL) approaches for detection tasks. By leveraging intrinsic image structure and coarse supervision, these methods aim to reduce dependence on dense voxel- or pixel-level labels while maintaining clinically acceptable performance. Table 3 summarizes representative studies, including dataset characteristics, supervision regime, and reported performance.

The limited availability of expertly annotated breast imaging datasets has driven the adoption of SSL and weakly supervised learning (WSL) approaches for detection tasks. By leveraging intrinsic image structure and coarse supervision, these methods aim to reduce reliance on dense voxel- or pixel-level labels while maintaining clinically acceptable performance. Table 3 provides an overview of representative studies, highlighting the employed model strategies, imaging modalities, specific tasks, datasets, and performance results.

In automated breast ultrasound (ABUS), MohammadiNasab et al. (29) proposed DATTR2U-Net, a double-attention recurrent residual U-Net trained with multi-task SSL proxy tasks, including rotation prediction and image reconstruction. On the TDSCABUS dataset, the model achieved a recall of 79.6% with 5.67 false positives per volume, representing an approximate 6% recall improvement over fully supervised baselines, despite using only 10% of voxel-level labels. This demonstrates SSL's potential to approximate full supervision in complex 3D ultrasound applications.

In mammography, Tardy et al. (24) developed a two-channel autoencoder integrating self- and weak supervision for abnormality detection and classification. Using INBreast and a private multi-vendor dataset, the model achieved an AUC of 0.86, a region-level true positive rate of 93%, and a pixel-wise F1 score of 64% for malignant masses. Notably, multi-vendor data improved model robustness, although external validation across larger, more diverse cohorts remains limited.

Alsuhbani et al. (30) addressed dense breast tissue localization using weakly supervised saliency mapping. Trained on 4,387 mammograms from the RSNA Breast Cancer Detection dataset, their CNN-based model achieved an accuracy of 75.3% and a Dice score of 75.4%. These results approached fully supervised U-Net performance while requiring only ∼15% of pixel-level annotations, highlighting the label efficiency of weak supervision.

Chang et al. (31) extended YOLOv8 with bi-level routing attention and bidirectional feature pyramid networks, incorporating SSL pretraining with rotation and cutout augmentations. On contrast-enhanced and standardized mammograms, the model achieved a precision of 99.3%, recall of 85.0%, and F1 score of 91.6% at an IoU threshold of 0.6. The high precision is clinically valuable for minimizing false positives, though sensitivity for subtle calcifications remains an area for improvement.

In MRI, Chen et al. (32) applied self-supervised rotation learning with a ResNet50 backbone to classify breast cancer vs. healthy tissue. On the Kaggle Breast Cancer MRI dataset (1,480 images), the model achieved an AUC of 95.8%, accuracy of 92.5%, sensitivity of 95%, and specificity of 90%, outperforming ImageNet-based transfer learning baselines. However, the relatively small and homogeneous dataset limits generalizability.

Across these studies, several patterns emerge. Proxy tasks such as rotation prediction and image reconstruction generally outperform contrastive learning variants in breast imaging, as they encourage the extraction of orientation-invariant and anatomically meaningful features. Integration of attention mechanisms—such as dual spatial–channel attention for ultrasound and bi-level routing attention for mammography—consistently improves detection and segmentation performance by enhancing feature discrimination. The use of image-level labels in weak supervision has enabled segmentation accuracy within approximately 10%–15% of fully supervised methods while dramatically reducing annotation requirements. Importantly, these models have maintained clinically acceptable false positive rates, which is essential for real-world deployment.

Despite these advances, several challenges remain. Most studies have relied on retrospective datasets, which may not fully capture the heterogeneity of clinical practice, including variations in scanner hardware, acquisition protocols, and patient demographics. Prospective clinical trials and external validation using multi-institutional datasets are essential to ensure robustness and generalizability. SSL proxy tasks may also struggle to capture rare or subtle pathologies, suggesting that combining multiple SSL tasks or integrating domain-specific priors could enhance sensitivity. Model interpretability remains a key barrier, as transparent and explainable outputs are necessary to secure clinician trust and regulatory approval. Additionally, AI systems must integrate seamlessly into existing imaging workflows to support, rather than increase, radiologists' cognitive workload.

Looking forward, several promising directions can be identified. Multi-modal SSL approaches that jointly leverage ABUS, mammography, and MRI could capture complementary information, improving diagnostic accuracy. Federated SSL frameworks provide a viable approach to train geographically distributed datasets while preserving patient privacy, potentially overcoming longstanding data-sharing restrictions. Advances in architecture design, including transformer-based models and refined contrastive learning variants, offer opportunities for richer feature extraction. The creation of standardized benchmarks and publicly available datasets specifically tailored to SSL and WSL in breast imaging will be critical for enabling rigorous, reproducible performance comparisons and accelerating methodological progress.

In summary, current evidence demonstrates that SSL and WSL methodologies can achieve diagnostic performance comparable to fully supervised models across diverse breast imaging modalities while substantially reducing annotation burden. To realize their full clinical potential, the field must prioritize external validation, enhance interpretability, conduct prospective trials, and ensure smooth workflow integration. Through coordinated efforts in algorithm design, clinical research, and systems integration, SSL-driven AI tools have the potential to transform breast cancer screening, providing scalable, cost-effective, and advanced diagnostic capabilities to a wider global population.

## 5 Transformers in breast imaging

In recent years, Transformer-based models have seen rapid adoption for breast lesion segmentation, detection, and classification. Both pure Transformer architectures and hybrid designs that integrate CNNs with self-attention mechanisms have demonstrated considerable promise in medical image analysis. Their primary advantage lies in the ability to capture fine-grained local texture information alongside global contextual relationships, a capability particularly relevant for breast imaging, where lesions often exhibit complex and heterogeneous appearances. Across mammography, ultrasound, and MRI, diagnostic interpretation benefits from models that can represent both localized structural patterns and broader anatomical context, enabling more accurate lesion characterization and supporting clinical decision-making.

### 5.1 Transformer-based breast lesion segmentation

Accurate segmentation of breast lesions is a fundamental prerequisite for effective computer-aided diagnosis and individualized treatment planning. Segmentation is challenged by anatomical variability, imaging artifacts, speckle or noise patterns, and the scarcity of high-quality annotated datasets. While traditional CNN architectures are effective at capturing local spatial features, they are inherently limited in modeling long-range dependencies. Transformer-based models have begun to address these limitations by incorporating global self-attention mechanisms, enabling the capture of contextual relationships across entire images or volumes. Table 4 summarizes representative peer-reviewed studies employing Transformer-based segmentation approaches across mammography, MRI, and ultrasound, highlighting consistent improvements over finely tuned CNN baselines.

**Table 4:** Transformer-based breast image segmentation studies.

Refs	Year	Model	Task	Imaging modality	Dataset	Performance
(40)	2022	Swin-SFTNet	Micro-mass segmentation	Mammography	CBIS-DDSM, INbreast	Dice Improvement: + 3.10% (CBIS-DDSM), + 3.81% (InBreast), + 3.13% (CBIS→InBreast)
(41)	2023	TrEnD (Transformer-based encoder–decoder)	Breast mass segmentation	Mammography	CBIS-DDSM (mix-frame), INbreast (mix-frame)	CBIS-DDSM: Dice: 92.20%, IoU: 85.81%; INbreast: Dice: 91.83%; IoU 85.29%
(42)	2023	TraBS	Fibroglandular tissue segmentation	MRI	200 internal, 40 external	Dice (Internal/External): 91.6% ± 6.7%/86.4% ± 8.1%, Surface Distance (mm):0.548 ± 2.195/0.584 ± 0.413
(43)	2022	RSTUnet-CR (Residual Swin Transformer U-Net with Consistency Regularization); Residual Swin Transformer encoder blocks; Dual-decoder	Tumor segmentation	ABUS	Proprietary ABUS: 84 480 frames from 256 subjects (1: 19 lesion/non-lesion ratio)	Acc: 79.57%, Dice: 60.43%, mIoU: 51.83%, Recall: 68.96%, HD95: 7.74 mm
(44)	2023	DSTransUFRRN	Lesion segmentation	Ultrasound	BUSI	Dice: 83.42%
(45)	2023	HCTNet	Lesion segmentation	Ultrasound	BUSI, BUS, Dataset B	BUSI: Dice:82% Acc:96.94%, Jaccard:71.84%, Recall:82.14%, Precision: 83.24%; BUS: Dice:84.13% Acc:98.49%, Jaccard:73.83%, Recall:83.19%, Precision: 88.5%; Dataset B: Dice:97.23% Acc:97.41%, Jaccard:94.63%, Recall:97.33%, Precision: 97.14%;
(46)	2024	ViT + UNet	Breast Tumor Segmentation	Ultrasound	BUSI	Dice: 75.84%; IoU: 62.92%; Precision: 79.01%; Recall: 78.82%, F1 score: 75.84%
(47)	2024	Swin-Net (Swin-T + RLM + HFM)	Breast tumor segmentation	Ultrasound	BUSI, BUS-B, BUS-O	BUSIS: Dice: 81.8%, Precision: 83.4%, Recall: 84.4%; BUS-B: Dice: 83.7%, Precision: 85.6%, Recall: 86.3%; BUS-O: Dice: 84%, Precision: 85.5%, Recall: 85%
(48)	2023	Faster Boundary-aware Transformer (FBAT): Boundary-wise Attention Block (BAB) in each transformer encoder layer, Reference-point-guided cross-attention in decoder for faster convergence	Breast lesion segmentation	Ultrasound, MRI	BUSI: 647 benign/malignant ultrasound images (512 × 512); BUSB: public ultrasound test set; Private BMRI: 1 200 MRI images	BUSI + BUSB: Dice 75.02%; IoU 65.37% (200 epochs); BMRI: Dice 89.69%; IoU 82.75% (200 epochs)

In mammography, early integration of Transformers has demonstrated substantial gains in delineating small and subtle lesions that CNNs often struggle to segment accurately. Zhao et al. (40) proposed Swin-SFTNet, which leverages the hierarchical Swin Transformer architecture for micro-mass segmentation. The model achieved Dice coefficient improvements of 3.10% on CBIS-DDSM and 3.81% on INBreast, with a notable 3.13% gain in cross-dataset testing when trained on CBIS-DDSM and evaluated on INBreast. Liu et al. (41) introduced Transformer-based encoder–decoder (TrEnD), optimized with mix-frame training, achieving Dice scores exceeding 92% and IoU values above 85% across both CBIS-DDSM and INBreast. These findings indicate that Transformer attention mechanisms enhance the ability to balance local texture modeling with global anatomical structure, a capability particularly beneficial for detecting minute or low-contrast lesions.

In MRI segmentation, the volumetric nature of the data and pronounced tissue heterogeneity create challenges that Transformers are particularly well-suited to address. Schmidt et al. (42) developed TraBS for fibroglandular tissue segmentation, reporting Dice scores of 91.6% ± 6.7% on internal datasets and 86.4% ± 8.1% on external datasets, alongside sub-millimeter surface distance errors (0.548 ± 2.195 mm internally, 0.584 ± 0.413 mm externally). These results demonstrate a high degree of robustness to variability in acquisition protocols, underscoring the potential of Transformer architectures for quantitative MRI analysis in diverse clinical settings.

In ultrasound imaging, where speckle noise, operator dependence, and heterogeneous acquisition protocols contribute to substantial domain shifts, Transformer-based approaches have been extensively explored in both pure and hybrid forms. Zhuang et al. (43) introduced the residual Swin Transformer U-Net with consistency regularization (RSTUnet-CR), incorporating residual Swin Transformer encoder blocks with a dual-decoder for segmentation and reconstruction. While achieving a moderate Dice score of 60.43% on large-scale ABUS datasets, the model demonstrated strong lesion recall (68.96%) and improved boundary preservation, attributes critical for surgical planning. Wang et al. (44) presented DSTransUFRRN, achieving a Dice score of 83.42% on the BUSI dataset. He et al. (45) developed HCTNet, a hierarchical cross-attention Transformer–CNN hybrid, which achieved exceptionally high Dice scores across three ultrasound datasets, including 97.23% on Dataset B, with balanced precision and recall above 97%. Zhang et al. (46) combined ViT with U-Net, yielding precision and recall values of approximately 79% each, though the Dice score of 75.84% suggests further optimization is possible. Li et al. (47) proposed Swin-Net, integrating relative location modeling and hierarchical fusion modules, maintaining Dice scores above 81% across multiple datasets.

Expanding into multimodal applications, Zhou et al. (48) introduced the faster boundary-aware transformer (FBAT), which incorporates boundary-wise attention and reference-point-guided cross-attention, applying it to both ultrasound and MRI. This approach achieved a Dice score of 75.02% on ultrasound datasets and 89.69% on MRI, while demonstrating accelerated convergence compared with conventional Transformer baselines.

Comparative analysis across these studies reveals several notable trends. Variants of the Swin Transformer architecture dominate both mammography and ultrasound research, likely due to their hierarchical token representation and robust multi-scale feature extraction. MRI-based Transformer segmentation, although less studied, shows strong generalization potential, indicating that further research and larger-scale validation could solidify their role in volumetric breast imaging. In ultrasound, performance variability across datasets highlights the persistent challenge of domain shifts, as models trained on curated or internal datasets often experience substantial degradation when evaluated externally. Nevertheless, Transformer-based models consistently outperform CNN-only counterparts, demonstrating their ability to integrate fine-grained spatial features with global contextual cues, which is crucial for accurate lesion delineation.

Transformer-based segmentation has emerged as a promising methodological direction in breast imaging, showing strong performance across multiple modalities. Their inherent flexibility supports both pure Transformer configurations and hybrid architectures that combine the global contextual modeling of self-attention with the fine-grained spatial sensitivity of convolutional networks. Moreover, their adaptability to multimodal pipelines—such as those integrating mammography, ultrasound, and histopathology—suggests considerable potential for advancing computer-aided diagnosis systems.

Future research priorities should include developing domain adaptation techniques to mitigate performance variability across institutions, establishing standardized multi-institutional benchmarks, and optimizing architectures to reduce computational costs without compromising accuracy. Equally important is the seamless integration of these models into clinical workflows to ensure outputs support radiologists' decision-making without introducing additional complexity. Addressing these considerations will be essential for translating Transformer-based approaches into scalable, clinically impactful solutions for breast imaging.

### 5.2 Transformer-based breast lesion detection

In recent years, ViTs and their hierarchical or cross-modal extensions have seen growing adoption for breast lesion detection. These models leverage self-attention mechanisms to capture both fine-grained local features and long-range contextual relationships, which are critical for identifying subtle lesions and complex tissue patterns. Table 5 summarizes key Transformer-based studies in this area, highlighting the diversity of architectural innovations and performance results across different imaging modalities.

**Table 5:** Transformer-based breast lesion detection studies.

Refs	Year	Model	Task	Imaging modality	Dataset	Performance
(49)	2022	Multi-View Vision Transformer	Breast Cancer Diagnosis	Mammography	949 cases (470 malignant, 479 benign/normal)	AUC: 81.8%
(50)	2023	TransReg (Cross-Transformer + Swin-T + Faster R-CNN)	Mass Detection & Auto-Registration	Mammography	DDSM, VinDr-Mammo	Recall @ 0.5 FP/image: 83.3% (DDSM), 79.7% (VinDr-Mammo)
(51)	2024	Transformer-Based Mammogram Classifier	Breast cancer detection (binary)	Mammography	DDSM, MIAS, INbreast, VinDr-Mammo	Acc:95.9%, AUC: 97.7%, Recall: 94.9%, Precision: 97.1%
(52)	2024	Dual-View Cross Attention with Swin Transformer	Breast cancer detection	Mammography	RSNA dataset	Acc: 81%; AUC: 87%
(53)	2024	Transferred-learning Deformable DETR with deformable attention modules on ResNet-50 backbone; decoder queries tuned (50, 75, 100, 125, 150)	Breast mass detection	Mammography	Pre-training: COCO 2017 (18 000 images, 91 classes); Fine-tuning: INBreast (410 images, MLO and CC views)	Best model (50 queries): mAP₅₀ = 0.68, mAP₅₀:₉₅ = 0.41
(54)	2025	Ensemble of Vision Transformer (Vit-L16) and CNN backbones (ResNet50, EfficientNetB1, ProDense block) with a stack-ensemble scheme	Breast tumor detection (benign vs. malignant)	Mammography	INbreast	Acc: 98.08%
(55)	2022	BUViTNet (Stage-wise ViT pre-trained on ImageNet + cancer cells)	Breast Lesion Detection	Ultrasound	BUSI, Mendeley, Mixed	AUC: 100% (Mendeley), 96.8% (BUSI), 93.7% (Mixed)
(56)	2022	HoVer-Trans (Anatomy-aware horizontal + vertical transformers)	ROI-Free Breast Cancer Diagnosis	Ultrasound	GDPH&SYSUCC, 2 others	AUC: 88.1%, Acc: 89.3%, Sensitivity: 83.6%, Precision:90.6%, Recall: 92.6%, F1: 91.6%
(57)	2024	Progressive Fine-Tuned ViT	Breast Lesion Detection	Ultrasound	BUS (780 images)	AUC: 92.1%, Acc: 94.49%
(58)	2025	DAMF-former (Dual-Modal Adaptive Mid-Term Fusion Transformer)	Axillary lymph node metastasis diagnosis	Ultrasound elastography (B-mode + shear-wave elastography)	Axillary UE scans from early breast cancer patients (*N* not specified)	Junior radiologist AUC improved from 0.807 to 0.883; inter-reader *κ* 0.805–0.895
(59)	2023	MMT (Multi-Modal Transformer)	Cancer Detection & 5-Year Risk Prediction	Mammography + Ultrasound	1.3 million exams	AUC: 94.3% for cancer detection AUC: 82.6% for 5-year risk
(60)	2025	Frozen large-scale pretrained vision-language models as foundational backbone, employing a frozen vision-language encoder plus a lightweight trainable classifier	Multimodal breast cancer prediction	Mammography & clinical EHR data	CBIS-DDSM; EMBED	CBIS-DDSM: AUC improved from 86.7% to 90.2%; test AUC from 0.80.3% to 83%; EMBED: AUC improved from 78% to 80.5%

In mammography, early Transformer models explored multi-view integration, leveraging the inherent symmetry and paired imaging protocols in breast screening. Chen et al. (49) introduced a Multi-View Vision Transformer that simultaneously processes craniocaudal and mediolateral oblique views, using cross-attention to align anatomical correspondences without explicit spatial registration. The model achieved an AUC of 81.8% on a moderately sized in-house dataset, performing comparably to CNN-based architectures such as DenseNet and EfficientNet. Its ability to learn view-invariant features without extensive preprocessing highlights a promising direction for multi-view mammographic AI.

Nguyen et al. (50) advanced this approach with TransReg, a hybrid architecture combining a Swin Transformer backbone, a cross-transformer registration module, and a Faster R-CNN detection head. By learning to spatially normalize the contralateral breast, TransReg enables detection on difference images rather than raw intensities, mirroring radiologists' comparative reading strategies. The model achieved recalls of 83.3% on DDSM and 79.7% on VinDr-Mammo at a low false-positive rate of 0.5 per image, outperforming both standard Faster R-CNN and Transformer-free baselines by 4%–6%. This work illustrates how Transformer-based attention mechanisms can be tailored to capture anatomical context unique to breast imaging, resulting in tangible gains in lesion localization.

The scalability of Transformer architecture was demonstrated by Shen et al. (51), who introduced the multi-modal transformer (MMT). Trained on 1.3 million screening exams, MMT integrates full-field digital mammograms and matches ultrasound volumes within a single model. It achieved an AUC of 94.3% for concurrent cancer detection and 82.6% for five-year risk prediction, outperforming ensemble CNN baselines by over five percentage points. This study highlights two core strengths of Transformers: their ability to integrate heterogeneous data modalities within a unified framework and their favorable performance scaling properties, where accuracy improves with increasing dataset size when representational bottlenecks are avoided.

In parallel, Transformer adoption in breast ultrasound has accelerated, driven by high frame rates, expanding public datasets, and growing use in dense breast populations. Ayana et al. (54) proposed BUViTNet, a ViT architecture pre-trained on ImageNet, then fine-tuned on cancer-cell histology patches before adaptation to ultrasound. This biologically inspired pretraining strategy yielded AUCs of 100% on the Mendeley dataset, 96.8% on BUSI, and 93.7% on a mixed dataset, outperforming standard CNNs by 3%–5% points. These results demonstrate the potential of multi-stage transfer learning to align general visual features with domain-specific tumor morphology, even in low-data regimes.

Addressing the challenge of ROI dependency in ultrasound, Mo et al. (55) developed HoVer-Trans, an anatomy-aware architecture that processes full ultrasound frames using separate horizontal and vertical self-attention streams. This directional bias enhances sensitivity to spiculated and irregular mass boundaries while suppressing false positives from acoustic shadowing and other artifacts. HoVer-Trans achieved an AUC of 88.1%, F1 score of 91.6%, and accuracy of 89.3% across three independent Chinese cohorts, substantially outperforming CNN counterparts in both sensitivity and precision.

Alruily et al. (56) demonstrated that Transformers can be effective in data-constrained settings through progressive fine-tuning. A vanilla ViT was first trained on natural images, then adapted to a thyroid ultrasound corpus, and finally fine-tuned on only 780 breast ultrasound images. Despite the limited dataset, the model achieved an AUC of 92.1% and accuracy of 94.5%, validating the effectiveness of domain-adaptive transfer learning in mitigating data scarcity and emphasizing the importance of task-aware pretraining pipelines.

Collectively, these studies reveal several recurring design principles underlying the success of Transformer-based breast lesion detection. Cross-view and cross-modal attention mechanisms enhance performance by integrating complementary information, whether across mammographic projections or between imaging modalities. Hierarchical and directional attention strategies, as in Swin Transformers and HoVer-Trans, enable broader contextual awareness while preserving spatial resolution, critical for detecting small, diffuse, or spiculated lesions. Multi-stage pretraining approaches grounded in biologically or anatomically relevant domains further improve robustness by accelerating convergence and enhancing generalizability. Compared with leading CNN architectures, Transformer models consistently yield 3%–6% gains in key metrics such as AUC, recall, and F1 score across diverse datasets, often without labor-intensive preprocessing steps like manual ROI annotations or bilateral registration, supporting greater automation and clinical applicability.

Despite these advances, several challenges remain. Many studies rely on retrospective, single-center datasets, limiting generalizability across scanners, populations, and clinical environments. Prospective clinical validation through blinded reader studies, real-world implementation trials, or integration into PACS/RIS infrastructure is essential for regulatory approval and clinical deployment. Model size and computational demands also pose practical constraints. Large-scale Transformers, such as ViT-L and Swin-B, perform well but may be impractical in low-resource or time-sensitive settings. Future work should explore lightweight Transformer variants, hybrid CNN–Transformer architectures, and model compression techniques for scalable adoption. Although attention mechanisms offer a pathway to model interpretability, few studies have systematically evaluated their transparency in clinical workflows. Incorporating attention heatmaps, saliency overlays, and formal explainability frameworks could foster clinician trust and meet emerging regulatory expectations for accountability and interpretability.

### 5.3 Transformer-based breast lesions classification

Recent advances in Transformer-based architectures have led to substantial improvements in breast lesion classification across mammography, ultrasound, and MRI. These models are particularly effective at capturing both local texture patterns and global contextual relationships, which are critical for distinguishing benign from malignant lesions. By integrating information across entire images or volumes, Transformers have the potential to enhance early cancer detection, reduce unnecessary biopsies, and streamline radiology workflows. Table 6 summarizes representative studies in this domain, highlighting the diversity of network designs, imaging modalities, and reported performance outcomes.

**Table 6:** Transformer-based breast lesion classification studies.

Refs	Year	Architecture	Task	Imaging modality	Dataset(s)	Performance
(61)	2022	Residual CNN + Transformer Encoder	Binary & Multiclass Classification	Mammography	CBIS-DDSM, DDSM	Acc: 100% (Binary), 95.8% (Multiclass)
(62)	2023	Ensemble CNN + Vision Transformer Encoder (ViT)	Binary & Multiclass Classification	Mammography	INbreast, Private annotated set	Acc: 98.58% (Binary), 97.87% (Multiclass); Private set: 97.16% (Binary), 89.4% (Multiclass)
(63)	2024	Swin Transformer	Four-category Breast Density Classification (BI-RADS)	Mammography	Small curated set	Acc: 74.96%
(64)	2024	LCVT-GR (Backbone + LCVTM + GRM)	Benign & Malignant Classification	Mammography	Mini-DDSM, CMMD	Mini-DDSM: AUC-ROC: 85.85%, AUC-PR: 65.76%; CMMD:AUC-ROC: 87.12%, AUC-PR: 89.03%;
(65)	2025	Dense-UMAF + DeiT (Dual-Track)	Classification of Masses & Microcalcifications	Mammography	CBIS-DDSM (Curated Subset)	Accuracy: 88.69%
(66)	2025	ViT-based	Benign & Malignant Classification	dynamic contrast-enhanced MRI	DCE-MRI dataset	Precision:80%, Recall:80%, F1 score: 80%, AUC: 80%
(67)	2022	VGGA-ViT: VGG attention vision transformer network combining a VGG-based CNN module (local feature extractor with SE block) and a ViT module (global relationship learner), ImageNet-pretrained	Benign & Malignant Classification	Ultrasound	Two BUS datasets: Dataset A (cross-validation); Dataset B (independent test)	Dataset A: acc: 88.71% ± 1.55%, recall: 90.735 ± 1.57%, specificity: 85.58% ± 3.35%, precision: 90.77% ± 1.98%, F1:90.73% ± 1.24%, MCC:76.34% ± 3.29%; Dataset B: acc: 81.72% ± 2.99%, recall: 64.45% ± 2.96%, specificity: 90.28%± 3.51%, precision: 77.08% ± 7.21%, F1:70.11% ± 4.25%, MCC:57.64%± 6.88%.
(68)	2025	Multimodal Sieve Transformer with ViT encoder (MMST-V); integrates UF-DCE MRI volumes, lesion characteristics, and clinical/geometrical data	Benign & Malignant Classification	Ultrafast dynamic contrast-enhanced MRI + clinical reports	240 patients; 987 lesions (280 benign, 121 malignant, 586 benign lymph nodes); 1 081 radiology reports	MMST-V: AUROC 0.928 ± 0.027; non-imaging only: AUROC 0.900 ± 0.045; imaging only: AUROC 0.863 ± 0.025
(69)	2024	Three ImageNet-pretrained Vision Transformer transfer-learning architectures evaluated on mammograms (Mendeley Data) and ultrasound (Mendeley Data & Kaggle), compared to ViT trained from scratch and CNN-based TL	Benign & Malignant Classification	Mammography and ultrasound	Mendeley Data mammogram dataset; Mendeley Data ultrasound dataset; Kaggle breast ultrasound dataset	AUC 1.0 ± 0 for both modalities, outperforming ViT from scratch and CNN-based transfer learning

In mammography, hybrid CNN–Transformer frameworks have demonstrated outstanding classification performance. Al-Tam et al. (61) combined a residual CNN with a Transformer encoder, achieving perfect binary classification accuracy (100%) and 95.8% accuracy for multiclass tasks on the CBIS-DDSM and DDSM datasets. Similarly, Al-Hejri et al. (62) employed an ensemble CNN with a ViT encoder, reaching 98.58% (binary) and 97.87% (multiclass) accuracy on INbreast, with slightly lower but still competitive results on a private dataset. These findings underscore the advantage of integrating CNN-based local feature extraction with the Transformer's global attention mechanism, enabling the capture of both fine-grained details and broader contextual patterns. For breast density classification, Tsai et al. (63) applied a Swin Transformer, achieving 74.96% accuracy and highlighting the persistent challenges posed by intra-class variability and limited annotated data.

Specialized Transformer architectures have also shown promise. Wu et al. (64) developed LCVT-GR, which incorporates local channel-wise ViT modules with a gating refinement mechanism, achieving AUCs of 85.85% on Mini-DDSM and 87.12% on CMMD. Paavankumar et al. (65) proposed a dual-track Dense-UMAF + DeiT pipeline to differentiate masses from microcalcifications, reaching 88.69% accuracy on a curated CBIS-DDSM subset. Beyond mammography, Wang et al. (66) compared multiple CNN and Transformer variants for dynamic contrast-enhanced MRI, reporting consistent but moderate performance across architectures (AUC ≈ 84%), suggesting that lesion enhancement patterns in MRI present distinct feature-learning challenges compared with the structural cues in mammography.

In ultrasound imaging, Transformer integration has similarly yielded advances. Qu et al. (67) developed VGGA-ViT, combining a VGG-SE CNN for local feature extraction with a ViT for global context modeling. On BUS Dataset A, the model achieved 88.71% accuracy and an F1-score of 90.73%, but performance dropped to 81.72% accuracy on an independent Dataset B, illustrating the domain shift problem in ultrasound. Ayana et al. (69) demonstrated that ImageNet-pretrained ViTs, when fine-tuned for breast imaging, can achieve perfect AUC (100%) for both mammography and ultrasound, outperforming CNN-based transfer learning and ViTs trained from scratch. These results emphasize the critical role of large-scale pretraining in overcoming data scarcity and improving generalization.

Multimodal approaches represent a promising frontier. Lokaj et al. (68) introduced MMST-V, which integrates ultrafast DCE-MRI with clinical and geometrical data using a multimodal sieve Transformer. This model achieved an AUC of 92.8% ± 2.7%, outperforming single-modality imaging (86.3%) and non-imaging inputs alone (90%). These findings highlight the diagnostic value of fusing anatomical, functional, and clinical data to generate richer, more comprehensive representations.

Collectively, the evidence indicates that hybrid CNN–Transformer pipelines excel in mammography by effectively combining local texture analysis with global spatial relationships, while multimodal fusion approaches hold promise for MRI-based diagnosis. Ultrasound classification remains highly susceptible to domain shifts, emphasizing the need for robust domain adaptation strategies. For clinical translation, future research should prioritize validation on large, diverse, and prospective datasets, and focus on integrating these models into radiology workflows in ways that enhance decision-making without increasing cognitive load.

### 5.4 Transformer-based multi-task learning in breast imaging

Recent advances in deep learning have markedly improved breast cancer detection and diagnosis across multiple imaging modalities, particularly mammography and ultrasound. These developments hold substantial clinical significance, as early and accurate identification of malignant lesions can meaningfully influence treatment strategies and patient outcomes. The studies summarized below illustrate how Transformer-based models, when combined with multi-task learning (MTL) frameworks, are being applied to address persistent challenges in breast imaging.

In 2021, Aly et al. (70) introduced a hybrid model integrating YOLO-v3 with ResNet and Inception-style Transformers for mass detection and classification in mammography using the INbreast dataset. Their method achieved a detection rate of 89.4%, a precision of 94.2%, and classification accuracies of 91.0% with ResNet and 95.5% with InceptionV3. By combining object detection with deep feature extraction, this approach proved particularly effective for high-resolution mammograms, where precise localization and characterization of masses are critical.

Building on this foundation, Su et al. (71) employed YOLOv5L6 enhanced with a LOGO Transformer to perform both detection and segmentation on the CBIS-DDSM and INbreast datasets. The model achieved a true positive detection rate of 95.7% and a mean average precision (mAP) of 65.0%. For segmentation, it reported an F1-score of 74.5% and an intersection over union (IoU) of 64.0%. This dual-task framework demonstrates the potential of Transformer-based models to simultaneously enhance spatial localization and boundary delineation, enabling more accurate lesion characterization in mammographic images.

In breast ultrasound, Rodriguez et al. (72) proposed a multi-task learning framework with a shared encoder and separate classification and segmentation heads. Four backbone architectures—VGG-16, ResNet-50, Swin Transformer V2 Tiny, and VMamba Tiny—were evaluated on a public BUS dataset. For segmentation, the USwin backbone achieved the highest mean IoU (85.59%), closely followed by VMamba (85.25%). For classification, VMamba attained the highest AUC (96.96%), precision (88.57%), and lowest false positive rate (4.4%), while ResNet Multi recorded the best true positive rate (94.87%), accuracy (92.31%), and F1-score (88.1%). This study represents the first application of the VMamba architecture to breast ultrasound and highlights the advantages of multi-task learning, which can optimize complementary tasks within a single model.

Despite these encouraging results, several challenges remain before widespread clinical adoption is feasible. Most datasets remain relatively small and lack diversity, limiting the generalizability of trained models across populations, scanners, and clinical settings. Transformer architectures often require substantial computational resources, which can hinder real-time use, particularly in resource-limited environments. Future research should focus on improving domain adaptation, employing advanced data augmentation strategies, and developing computationally efficient model designs to facilitate scalable, real-world deployment.

## 6 Hybrid technologies in breast imaging

Recent advancements in breast imaging highlight the substantial potential of combining SSL with Transformer architectures. These hybrid models effectively address persistent challenges, including lesion variability, limited annotated data, and heterogeneous tissue appearances, across modalities such as ultrasound, MRI, and mammography. Table 7 provides a comprehensive overview of key studies, summarizing architectures, imaging modalities, datasets, tasks, and reported performance outcomes. This section reviews these developments by task category, highlighting methodological strengths, comparative results, and clinical implications.

**Table 7:** Hybrid -based breast imaging studies.

Refs	Year	Model	Task	Modality	Dataset	Performance
(73)	2023	CSwin-PNet (CNN + Swin Transformer Pyramid Network)	Breast Lesion Segmentation	Ultrasound	Dataset1 (High-quality BUS images), Dataset2 (Lower-quality BUS images)	Dice: 87.25% (Dataset1), 83.68% (Dataset2), IoU: 78.61% (Dataset1), 75.11% (Dataset2)
(74)	2023	HEAT-Net	Segmentation	Ultrasound	BUSI, DDTI, TN3k, CAMUS	Dice: 74.1% (BUSI), 82.7% (DDTI), 89.5% (TN3k), 94% (CAMUS)
(75)	2024	GED-Net	Segmentation	Ultrasound	DatasetB, DDTI, OASBUD, BUSI	DatasetB: Acc:98.4%, IoU:68%, Recall:0.78.3%, Dice:77.4%, Precision:74.4%; DDTI: Acc:94%, IoU:62.2%, Recall:79.1%, Dice:74%, Precision:73.1%; OASBUD: Acc:96.7%, IoU:55.7%, Recall:72%, Dice:67.9%, Precision:74.3%; BUSI: Acc:96.1%, IoU:69.4%, Recall:80.1%, Dice:78.1%, Precision:74.3%
(76)	2024	Human Learning Paradigm Network	Segmentation	Ultrasound	Local (600 images, 30 patients), BUSI, DatasetB	Best variant: 0.76% ↑ Dice, 3.14 mm ↓ HD vs. TransUNet Public dataset: 0.42% ↑ Dice, 5.13 mm ↓ HD vs. TransUNet, Training time ↓ 31.25%
(77)	2024	BGRD-TransUNet	Segmentation	Ultrasound	BUSI, DatasetB	BUSI: DSC: 71.54%, IoU: 69.76%, Recall:74.27%, Precision: 72.96%, Acc: 97.52%; DatasetB: DSC: 75.27%, IoU: 64.92%, Recall: 91.41%, Precision:69.15%, Acc: 97.38%;
(78)	2025	FET-UNet (CNN + Transformer)	Segmentation	Ultrasound	BUSI/UDIAT/BLUI	Dice: 82.9% (BUSI), 88.9% (UDIAT), 90.1% (BLUI)
(79)	2025	3D segmentation network (i.e., DST-C)	Segmentation	ABVS Ultrasound	Private ABVS + TDSC-ABUS 2023	Dice: 73.65%, IoU: 61.10%, Sensitivity: 91.67%, HD: 23.23 mm
(80)	2022	TR-IMUnet (Transformer + Multi-scale CNN)	Tumor Segmentation	DCE-MRI	Private dataset (clinical cases)	Dice: 96.25%, IoU: 90.55%, Sensitivity: 96.26%, PPV: 94.92%
(81)	2025	PLHN (Prototype Learning Guided Hybrid Network)	Tumor Segmentation + Diagnosis	DCE-MRI	Public & Private DCE-MRI datasets	AUC: 66.6%, Acc: 63.8%, Precision: 64.0%, Recall: 33.5%, F1 Score: 41.3%, Dice: 85.6%
(82)	2025	Hybrid Transformer U-Net (HTU-Net)	Breast Mass Segmentation	Mammography	CBIS-DDSM & INbreast	CBIS-DDSM: Dice: 93.5%, IoU: 87.41%, Acc: 98.43%, Sensitivity: 94.01%, Specificity: 97.18%; INbreast: Dice: 92.14%, IoU: 86.08%, Acc: 95.16%, Sensitivity: 93.89%, Specificity: 95.11%
(83)	2025	Hybrid CNN + Transformer: CNN encoder (ResNet/EfficientNet) for local features → transformer blocks for global context → U-Net-style decoder for mask refinement	Breast tumor segmentation	Mammography	INbreast	Acc: 89.40%, Dice: 76.50%, mIoU:73.%, HD95: 4.80 mm
(84)	2023	Dual-input CNN + GAP-guided Attention Loss	Benign & Malignant Classification	Ultrasound	BUSI & BUSC	BUSI: Acc: 98.1%, Precision: 98.3%, Recall: 98.2%, F1 Score: 98.2%; BUSC: Acc: 97.9%, Precision: 97.5%, Recall: 98.1%, F1 Score: 97.8%
(28)	2023	HoVer-Transformer	ROI-Free Breast Cancer Diagnosis	Ultrasound	GDPH & SYSUCC (2,405 images)	Acc: 89.3%, AUC: 92.4%, Sensitivity: 92.6%, Specificity: 83.6%
(85)	2024	CNN + Multi-scale Transformer (Ensemble)	Normal, Benign, Malignant Classification	Ultrasound	BUSI	Acc: 98.70%, F1 Score: 98.72%
(86)	2024	Dynamic Pooling + Hybrid ViT-CNN	Benign & Malignant Classification	Ultrasound	BUS sequences	Acc: 93.78%
(87)	2024	PolyBreastVit: hybrid model combining PolyNet (multi-scale CNN) for detailed local feature extraction and Vision Transformer for global context	Three-way classification (benign/malignant/normal)	Ultrasound	880 high-definition ultrasound images from 500 women (ages 25–75); three classes; extensive preprocessing and augmentation	Overall acc: 98%; benign precision/recall: 98%/98%; malignant precision/recall: 96%/96%; normal precision/recall: 100%/100%; outperforms VGG-16, Inception V3, ResNet-50 across accuracy, precision, recall, F1, AUC
(88)	2025	CNN-Transformer + Segmentation Knowledge	Benign & Malignant Classification	Ultrasound	Breast & Thyroid datasets	Dice: 83.62%, AUC: 95.36%;
(89)	2025	C-TUNet (CNN + Transformer)	Benign & Malignant Classification	Ultrasound	BUSI	Acc: 96.7%, AUC: 97.1%, F1 Score: 96.5%
(90)	2025	CNN_ViT (Hybrid CNN + ViT)	Benign & Malignant Classification	Ultrasound	KAUH-BCUSD (6,159 images, 5,000 cases)	Acc: 95.12%, Recall: 97.54%, F1 Score: 95.24%
(28)	2022	Semi-supervised ViT + ATS	Benign & Malignant Classification	Ultrasound & Histopathology	BUSI & BreakHis	BUSI: Acc: 96.1%, AUC: 97.2%; BreakHis: Acc: 95.8%, AUC 96.9%
(91)	2024	Pyramid Transformer (PTr) + SAM	Benign & Malignant Classification	Mammography	INbreast	Acc: 99.96%, AUC: 99.98%
(92)	2024	HybridMammoNet: hybrid CNN–ViT with cross-view transformer layer linking ResNet18/VGG16 feature maps before pooling	Benign & Malignant Classification	Mammography	CBIS-DDSM	AUC:80%; F1-score: 65%
(93)	2025	Hybrid Transformer	Benign & Malignant Classification	Mammography	1,200 paired exams from 3 sites	Acc: 90.80%, Sensitivity: 90.80%, Precision: 90.80%, Specificity: 90.88%, F1 Score: 90.95%, AUC: 92.58%
(94)	2025	Hybrid CNN + ViT framework (local feature extractor *via* CNN, global context *via* Vision Transformer); compared with DenseNet, Inception, SE-ResNet, XceptionNet	Benign & Malignant Classification	Mammography	CLAHE-enhanced mammograms from Kaggle (balanced benign/malignant)	CNN + ViT: acc:90.1%; XceptionNet: acc:100% (likely overfitting)
(95)	2023	Dual-Input Transformer	pCR Prediction (NAC Response)	Ultrasound	484 cases from two clinical centers	Acc: 93.9%, AUC: 96%, F1 Score: 92.7%
(96)	2024	EPTM (Efficient CNN + Vision Transformer + Choquet Integral Fusion)	Malignancy Prediction	Ultrasound	UDIAT BUS, Baheya Hospital	AUC: 93.2% (UDIAT), 98% (Baheya)
(97)	2023	Feature extraction *via* SEResNeXt; attention-based classification with Swin Transformer	Breast cancer detection (benign vs. malignant)	MRI	Breast MRI scans (dataset size and source not specified)	Acc: 96%; F1 score: 61%
(98)	2024	Swin Transformer + CNN	Slice Selection + Tumor Diagnosis	Ultrasound	NTUH (807 patients)	Slice Selection: Top-1 Acc: 74.35%, Top-5 Acc: 97.27%; Diagnosis: Acc: 79.85%, Sensitivity: 80.13%, Specificity: 79.80%, AUC: 86.41%
(99)	2024	Spatio-Temporal Memory Net	Needle Tracking & Segmentation	Ultrasound	Real-time biopsy video (11 patients)	Dice: 81.7%, IoU: 73.1%, Precision: 86.3%, Recall: 80.3%, F1 score: 83.2%
(100)	2024	ACSNet (UNet-based with DSAModule, Gate Units, Channel Attention)	Segmentation and Classification	Ultrasound	Two publicly available BUS datasets	Segmentation: Dice: 84.90%, Jaccard: 78.62%, 95HD: 13.04 mm, ASD: 3.45 mm; Classification: Acc: 94.44%, Precision: 94.61%, Recall: 93.86%
(101)	2024	YOLOv4 backbone for mass detection coupled with a ViT transformer for classification	Breast mass detection & benign/malignant classification	CESM & FFDM Mammography	INbreast; CDD-CESM	Detection mAP: 98.69% (INbreast), 81.52% (CE-CESM), 71.65% (DM-CESM); Classification Acc: 95.65% (INbreast), 97.61% (CE-CESM), 80% (DM-CESM)

### 6.1 Segmentation

In ultrasound segmentation, hybrid models such as CSwin-PNet and HEAT-Net have demonstrated substantial improvements in Dice scores across multiple datasets, including both high-quality and lower-quality ultrasound images (Table 7) (73, 74). The integration of multi-scale Transformer blocks within U-Net frameworks enhances lesion boundary delineation, effectively addressing challenges posed by lesion heterogeneity and noisy imaging conditions. For volumetric data, Liu et al. (79) extended these concepts to 3D segmentation of automated breast volume scans, achieving respectable sensitivity but highlighting persistent difficulties in boundary precision, with an HD95 of 23.2 mm. These findings indicate that, despite the representational power of Transformers, accurately capturing fine-grained 3D lesion morphology remains a challenge.

In dynamic contrast-enhanced MRI, hybrid CNN–Transformer models such as TR-IMUnet achieve exceptional segmentation performance (80), illustrating the benefit of combining local convolutional feature extraction with global attention mechanisms. However, models addressing multi-task segmentation and diagnosis, exemplified by PLHN (81), reveal the complexity of jointly optimizing segmentation accuracy and malignancy prediction. Notably, PLHN exhibits reduced diagnostic recall despite solid Dice scores, underscoring the trade-offs inherent in multi-objective learning within breast MRI.

In mammography, Transformer-augmented U-Net architectures such as HTU-Net consistently outperform pure CNN models on benchmarks including CBIS-DDSM and INbreast, achieving Dice scores above 92% and accuracies exceeding 95% (82). Nonetheless, mammographic segmentation remains challenged by tissue overlap and dense breast patterns, with some models demonstrating reduced precision metrics (83). These limitations indicate that further innovations in network architecture and data augmentation strategies are needed to enhance spatial localization and fully exploit the potential of hybrid approaches.

### 6.2 Classification

The classification of breast lesions has similarly benefited from SSL–Transformer hybrid models, which use large unlabeled datasets for pretraining and capitalize on Transformers' capacity to integrate global contextual information. In ultrasound, classifiers such as PolyBreastVit and C-TUNet have demonstrated impressive accuracies exceeding 95%, accompanied by high precision and recall across benign, malignant, and normal classes (87, 89). These results indicate that multi-scale convolutional backbones combined with Transformer layers can robustly discriminate complex tissue patterns, even in heterogeneous imaging environments.

In mammography, pyramid Transformer architectures paired with masked self-attention mechanisms have achieved near-perfect benign vs. malignant classification (91). However, other hybrid frameworks report more variable AUC scores, with some as low as 80% (Table 7) (92). This variability highlights the critical influence of data quality, preprocessing pipelines, and the importance of standardized benchmarks to ensure reproducibility and generalizability in classification tasks.

While ultrasound classification approaches generally achieve high sensitivity and specificity, MRI-based classification performance remains relatively lower in some studies (97), suggesting that further refinement is needed to capture subtle malignant signatures within volumetric and functional imaging data. Incorporating multimodal data fusion and interpretability mechanisms represents a promising strategy to enhance accuracy and clinical applicability.

### 6.3 Detection

In breast lesion detection, hybrid SSL–Transformer models have demonstrated strong performance across both mammography and ultrasound modalities. For example, YOLOv4 combined with a ViT backbone achieved high mean average precision on full-field digital mammography and contrast-enhanced spectral mammography datasets (101), performing comparably to leading CNN-based detectors under similar evaluation conditions. In ultrasound, hybrid detection models have shown precise lesion localization and classification, with reported accuracies exceeding 97% (84). These results indicate that integrating Transformer-based attention with established detection backbones enhances spatial awareness and reduces false positives, improving overall detection reliability.

Despite these advances, real-time clinical deployment remains constrained by computational complexity and latency. The development of lightweight Transformer blocks and optimized attention mechanisms is essential to enable rapid inference without compromising accuracy, particularly in resource-limited environments. Additionally, the lack of standardized evaluation protocols across diverse, multi-center datasets limits comprehensive assessment of model generalizability. Addressing these challenges will be critical to translating hybrid SSL–Transformer detection models into scalable, clinically viable tools.

### 6.4 Multi-task frameworks and prognostic prediction

Hybrid SSL–Transformer models have also been extended to multi-task frameworks that simultaneously address segmentation, classification, and prognostic prediction. Models such as PLHN demonstrate the feasibility of combining lesion delineation with malignancy assessment, though diagnostic recall in some cases remains suboptimal (81). In contrast, Transformer-based approaches for predicting pathological complete response following neoadjuvant chemotherapy have achieved high accuracy and AUC (95), indicating that attention mechanisms can effectively capture imaging biomarkers associated with treatment response.

Multi-task learning in breast imaging requires careful architectural design to balance competing objectives and avoid performance trade-offs. Additionally, the clinical interpretability of these complex models is essential for adoption. Incorporating explainability techniques, such as attention rollout or Grad-CAM, can provide insights into model decision-making and help radiologists understand the rationale behind predictions, thereby supporting trust and facilitating integration into clinical workflows.

### 6.5 Synthesis and future directions

The studies reviewed collectively demonstrate that SSL pretraining accelerates model convergence and enhances robustness across diverse breast imaging modalities, while Transformer architectures contribute essential global context modeling and multi-scale feature integration (Table 7). Despite these advances, several research gaps remain. Standardized multi-center, multi-modality benchmark datasets are limited, restricting objective comparisons and impeding clinical translation.

Future research should focus on developing unified SSL frameworks that are adaptable across imaging types and incorporate domain adaptation strategies to mitigate cross-center variability. The high computational demands of Transformer architectures also highlight the need for efficient attention mechanisms to enable real-time, point-of-care deployment. Equally important is the integration of interpretability directly within model architectures, ensuring that decision-making processes are transparent and fostering clinician trust. Addressing these challenges through collaborative, interdisciplinary efforts will be critical to realizing the full potential of SSL–Transformer hybrid models in breast imaging, paving the way for scalable, robust, and clinically actionable AI solutions.

## 7 Discussion

### 7.1 Key research findings

The recent evolution of AI in breast imaging has been marked by a decisive shift toward SSL and Transformer-based architectures, fundamentally reshaping approaches to segmentation, classification, and detection tasks. The studies reviewed consistently demonstrate SSL's capacity to leverage large quantities of unlabeled imaging data, reducing reliance on extensive manual annotation while maintaining—or even enhancing—performance in downstream tasks. This is particularly valuable in breast imaging, where annotating small, heterogeneous lesions is time-consuming and prone to inter-observer variability.

In segmentation, SSL approaches particularly those integrating context restoration, contrastive pretraining, or pseudo-label refinements show notable gains in delineating lesion boundaries in challenging modalities such as ultrasound and MRI. Contrastive learning methods effectively capture discriminative features with relatively small labeled datasets, though they are sensitive to augmentation strategies and may underperform in the presence of high imaging variability. Masked autoencoders excel at reconstructing missing image regions and have demonstrated strong performance in mammography; however, they typically require larger unlabeled datasets and longer training times. Generative SSL approaches, such as GAN- or diffusion-based pretext tasks, offer potential for data augmentation and representation learning, though their clinical realism and stability remain limited. Collectively, these findings suggest that the optimal SSL strategy is likely modality- and dataset-dependent.

Classification tasks similarly benefit from SSL-pretrained encoders, particularly when combined with domain-specific augmentation strategies that replicate real-world imaging variability. The effectiveness of SSL in classification depends critically on aligning pretext task design with the distinct imaging characteristics of each modality.

Transformer-based architectures have emerged as equally transformative, providing powerful mechanisms to model global dependencies and multi-scale contextual relationships. These capabilities are vital for detecting microcalcifications, subtle spiculations, and complex lesion morphologies. Evidence indicates that ViTs offer strong global feature modeling but are computationally intensive and less robust on small datasets. Swin Transformers, with hierarchical window-based attention, improve scalability and efficiency, making them more suitable for high-resolution breast imaging tasks. Hybrid CNN–Transformer models strike a balance by leveraging CNNs for local texture extraction while using Transformers for global reasoning, often achieving competitive accuracy with reduced computational burden. Clinically, hybrid models may represent the most practical compromise, though prospective validation remains limited.

According to the reviewed literature, several converging trends are apparent. Researchers are increasingly focused on reducing annotation requirements through weak or scribble supervision, enhancing model interpretability via attention visualization, and incorporating multimodal data to overcome the limitations of single-modality analysis. These innovations are progressively aligning algorithmic performance with clinical priorities, including robustness across diverse patient populations and imaging platforms, and the ability to deliver reproducible results under varied acquisition conditions.

### 7.2 Challenges and limitations

SSL and Transformer-based architectures offer transformative potential in breast imaging; however, several recurring challenges currently impede their widespread clinical adoption. One of the most significant hurdles is the substantial computational demand of Transformer models. Their quadratic complexity relative to input size necessitates extensive memory and processing power, limiting real-time inference in clinical settings without access to high-performance GPUs or advanced hardware. Techniques such as sparse attention, token pruning, and hybrid CNN–Transformer architectures offer pathways to reduce computational complexity, yet these approaches require rigorous validation within clinical workflows to ensure diagnostic accuracy is preserved, particularly when deployed on standard hospital equipment.

The effectiveness of SSL critically depends on the design of pretext tasks tailored to specific breast imaging modalities. Inadequate task selection can produce suboptimal feature representations that fail to capture clinically relevant patterns, undermining downstream performance. Furthermore, SSL requires access to large, diverse unlabeled datasets, which are often constrained by privacy regulations and institutional barriers, limiting model robustness and generalizability.

Domain shifts arising from variability in imaging protocols, scanner manufacturers, and patient demographics remain a formidable challenge. Many studies relied on relatively small, single-center datasets without external validation, restricting generalizability. Standardized federated learning frameworks could address these issues by enabling collaborative model training without compromising patient privacy. Concurrently, the development of diverse, harmonized datasets reflecting real-world variability, alongside rigorous external validation, is essential to ensure fairness and minimize bias.

Interpretability remains a persistent limitation. While Transformer attention maps can highlight influential regions, their clinical relevance is often ambiguous. CNN-based saliency methods, such as Grad-CAM and SHAP, face similar challenges. The absence of standardized visualization frameworks undermines clinician trust and complicates regulatory approval, as agencies such as the FDA and EMA require both explainability and demonstrable safety.

Reproducibility and clinical applicability are further constrained by the predominance of retrospective, single-center studies. Annotation quality was inconsistently reported, with some studies relying on a single annotator or omitting details altogether. Only a minority of studies provided open-source code or pretrained models, restricting transparency and independent verification. Evaluation metrics were often incompletely reported, with some studies presenting only a single measure and omitting confidence intervals or statistical comparisons. Prospective, multi-institutional clinical trials embedding AI tools into real-world workflows remain scarce, limiting insights into their impact on diagnostic efficiency, clinician confidence, and patient outcomes.

Collectively, these limitations highlight the critical need for coordinated data sharing, standardized evaluation metrics, reproducibility practices, and multi-site prospective validation to ensure that SSL and Transformer-based methods can transition from promising research tools to clinically reliable systems.

### 7.3 Strategies for clinical translation and integration

Successful clinical translation of AI models, including Transformer architectures and SSL frameworks, requires more than high technical performance. It demands careful alignment with clinical workflows and the real-world needs of radiologists, oncologists, and multidisciplinary healthcare teams. AI tools that deliver actionable, interpretable outputs and integrate seamlessly with Picture Archiving and Communication Systems (PACS) and electronic health records have the highest potential for adoption. Co-designing AI interfaces with clinicians is essential to ensure these tools augment, rather than disrupt, diagnostic workflows and align with established clinical reasoning patterns.

Interpretability is pivotal for building clinician trust and facilitating regulatory approval. Recent strategies advocate combining attention maps with uncertainty quantification and counterfactual explanations to produce clinically meaningful insights. These approaches require validation in breast cancer imaging, emphasizing diagnostically relevant features such as lesion morphology and tissue heterogeneity that resonate with radiologists. Embedding explainable AI frameworks directly into user interfaces allows clinicians to verify AI suggestions, mitigating both skepticism and overreliance.

Beyond interface design, minimizing workflow disruption and optimizing computational efficiency to enable near real-time inference are critical. Techniques such as edge computing, hardware acceleration, and model compression can overcome latency and resource constraints, facilitating deployment even in resource-limited settings. Addressing interoperability challenges posed by heterogeneous hospital IT systems and ensuring compliance with data standards are also essential for scalable integration. Ongoing clinician education and training in AI capabilities and limitations further promote appropriate adoption. Incorporating patient perspectives and addressing ethical concerns—such as bias and informed consent—are increasingly recognized as integral to successful integration.

Embedding AI models into prospective, multi-center clinical trials is necessary to rigorously evaluate their impact on diagnostic accuracy, workflow efficiency, clinician confidence, and patient outcomes. Such evidence is foundational for informing regulatory decisions and reimbursement policies. Establishing standardized protocols for continuous monitoring, quality assurance, and post-deployment model updates ensures sustained safety and performance. Robust privacy and data governance frameworks are equally imperative. Federated learning and differential privacy offer promising strategies for collaborative model training without sharing raw patient data, effectively addressing ethical and legal constraints. Successful clinical translation depends on integrating these privacy-preserving approaches with strong cybersecurity measures and transparent data stewardship, fostering trust among clinicians and patients alike.

### 7.4 Future research directions

To fully realize the potential of AI in breast cancer imaging, future research should strategically address challenges related to computational efficiency, robustness, interpretability, and clinical validation. Advanced model compression techniques, including knowledge distillation, low-rank factorization, and pruning, tailored specifically for medical imaging data, could reduce computational demands while preserving diagnostic accuracy. Further development of hybrid architectures that combine CNNs' proficiency in local feature extraction with transformers' capacity for global context modeling represents a promising avenue. Dynamic inference strategies, which adaptively allocate computational resources based on image complexity, may optimize efficiency in real-time clinical environments but require validation in prospective workflows.

Robustness remains a critical barrier to clinical deployment. Research should expand domain adaptation methods, including adversarial training and contrastive learning, with rigorous evaluation across diverse datasets that reflect broad imaging protocols and patient demographics. Federated learning approaches also require refinement to handle inter-institutional heterogeneity, employing communication-efficient algorithms and personalized model aggregation to maintain diagnostic reliability while safeguarding patient privacy. Building large, diverse, and harmonized datasets with standardized acquisition and annotation protocols, coupled with comprehensive external validation, will be essential to mitigate domain shifts and reduce bias.

Interpretability may benefit from multi-layered frameworks that link pixel-level explanations with higher-level clinical feature representations, enhancing alignment with established medical knowledge. Incorporating uncertainty quantification alongside interpretability techniques could enable clinicians to assess confidence in AI outputs, fostering more informed decision-making. Expanding multi-modal integration beyond imaging to include pathology, genomics, and clinical data may yield richer, personalized diagnostic and prognostic insights, better reflecting the complexity of breast cancer.

Large-scale, prospective, multi-center clinical trials are urgently needed to evaluate not only diagnostic accuracy but also the impact of AI tools on clinical workflow, radiologist confidence, and patient outcomes. Such evidence will be critical for regulatory approval and incorporation into clinical guidelines. Ethical principles should be embedded throughout both research and deployment, including ongoing bias monitoring, transparent documentation of model updates, and rigorous data governance frameworks to protect patient rights and ensure equitable access to AI benefits.

Finally, research should explore the dynamics of human–AI interaction, examining how clinicians engage with AI tools, how workflows evolve, and the implications for healthcare disparities and access. Incorporating socio-technical frameworks will be essential to ensure AI innovations translate into practical, equitable, and sustainable improvements in breast cancer care.

## 8 Conclusion

This study reviews the emerging potential of SSL and transformer-based architectures in breast imaging, demonstrating their ability to enhance performance across lesion detection, classification, and segmentation tasks, particularly in scenarios with limited annotations. These approaches offer notable advantages in label efficiency, cross-modality adaptability, and robustness under data-scarce conditions. However, fully realizing their clinical impact depends on addressing persistent challenges, including the limited availability of large and diverse datasets, vulnerability to domain shifts, incomplete assessments of fairness and interpretability, high computational demands, and the absence of prospective validation frameworks.

Strategic directions for future research include the development of federated, vendor-agnostic SSL frameworks to enable privacy-preserving pretraining, along with parameter-efficient adaptation techniques for deployment in diverse clinical environments. Integrating AI tools within human-in-the-loop workflows can build clinician trust, while establishing standardized, bias-audited benchmarks will support reproducibility and fairness. Robust uncertainty quantification and continual learning protocols could further facilitate safe adaptation to evolving imaging technologies and heterogeneous patient populations.

Bridging the gap from promising research prototypes to clinically reliable tools will require coordinated multi-institutional collaboration, rigorous external validation, prospective clinical trials, and strong ethical oversight. Such concerted efforts are essential to ensure that SSL and transformer-based innovations meaningfully enhance the accuracy, efficiency, and equity of breast cancer care.
